# Supplementary material for: Malaria blood stage infection suppresses liver stage infection via host-induced interferons but not hepcidin
Source: Nat Commun. 2024 Mar 7;15:2104. doi: 10.1038/s41467-024-46270-3 (PMC10920859; doi:10.1038/s41467-024-46270-3)
Supplement: Supplementary file 1 — Supplementary Information [file 41467_2024_46270_MOESM1_ESM.pdf]

Supplementary Information

Malaria blood stage infection suppresses liver stage infection via host-induced Interferons but not hepcidin

Hardik Patel[1], Nana K. Minkah[1,2], Sudhir Kumar[1], Gigliola Zanghi[1], Antonino Schepis[1], Debashree Goswami[1], Janna Armstrong[1], Biley A. Abatiyow[1], Will Betz[1], Laura Reynolds[1], Nelly Camargo[1], Amina A. Sheikh[1], Stefan H.I. Kappe*[1,2,3]

[1]Center for Global Infectious Disease Research, Seattle Children's Research Institute, Seattle, WA, USA

[2]Department of Pediatrics, University of Washington, Seattle, WA, USA

[3]Department of Global Health, University of Washington, Seattle, WA, USA

*Correspondence: Stefan H.I. Kappe: stefan.kappe@seattlechildrens.org

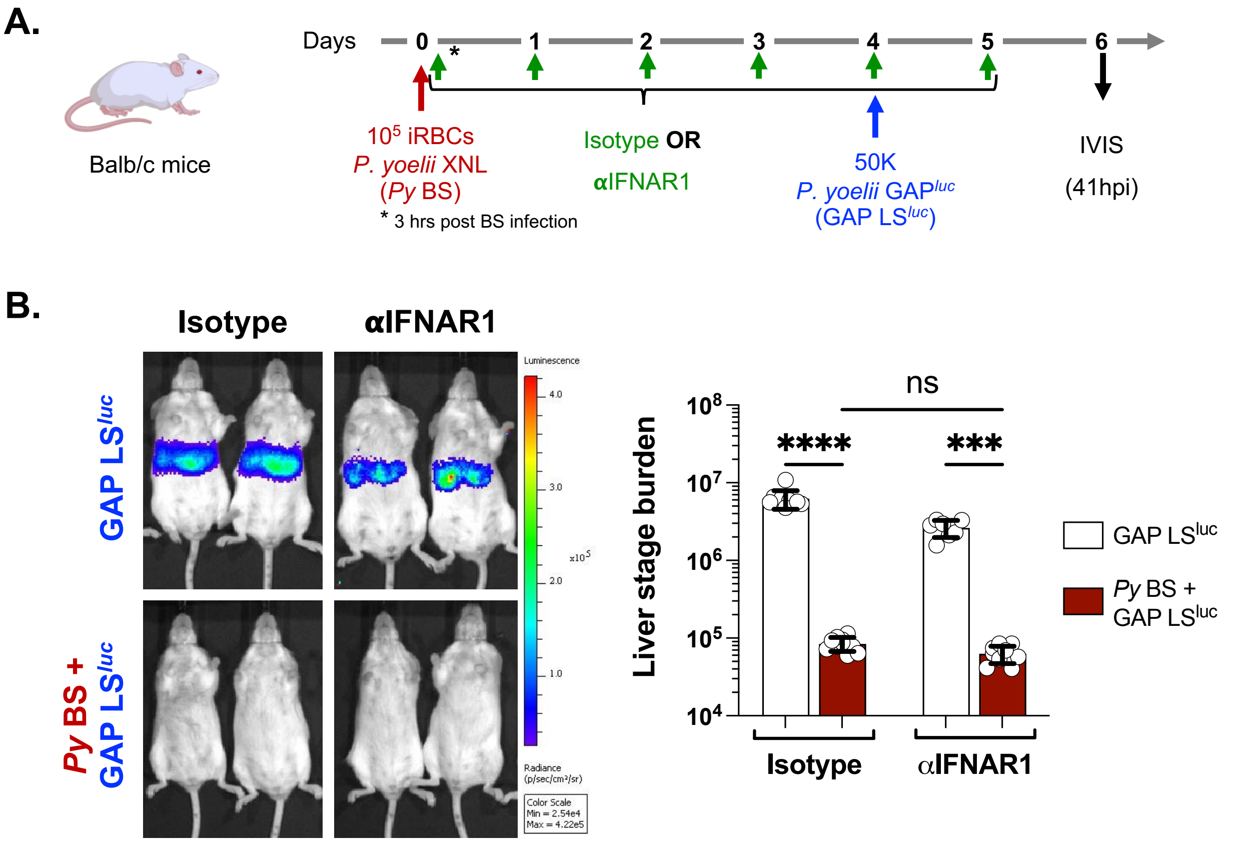

Supplementary Figure 1: ***P. yoelii BS infection does not suppress LS infection via type I IFN signaling.*** **(A)** Control (GAP LS^{luc}) and *Py* BS infected (*Py* BS + GAP LS^{luc}) mice were treated with Isotype or anti-IFNAR1 antibodies as shown in the scheme and were infected with 50,000 *Py* GAP^{luc} sporozoites on day 4 post BS infection. **(B)** Parasite liver load as measured by IVIS at 41 h after sporozoite injection and represented as total flux (p/s). Two-way ANOVA with Tukey's multiple comparison test for comparing groups with two variables. ****P<0.001, ****P<0.0001.* Results are combined and represented as means ± SD from two independent experiments (N = 7-10 mice per group). Source data are provided as a Source Data file. The graphical illustration of the mouse in (A) was made using BioRender.com.

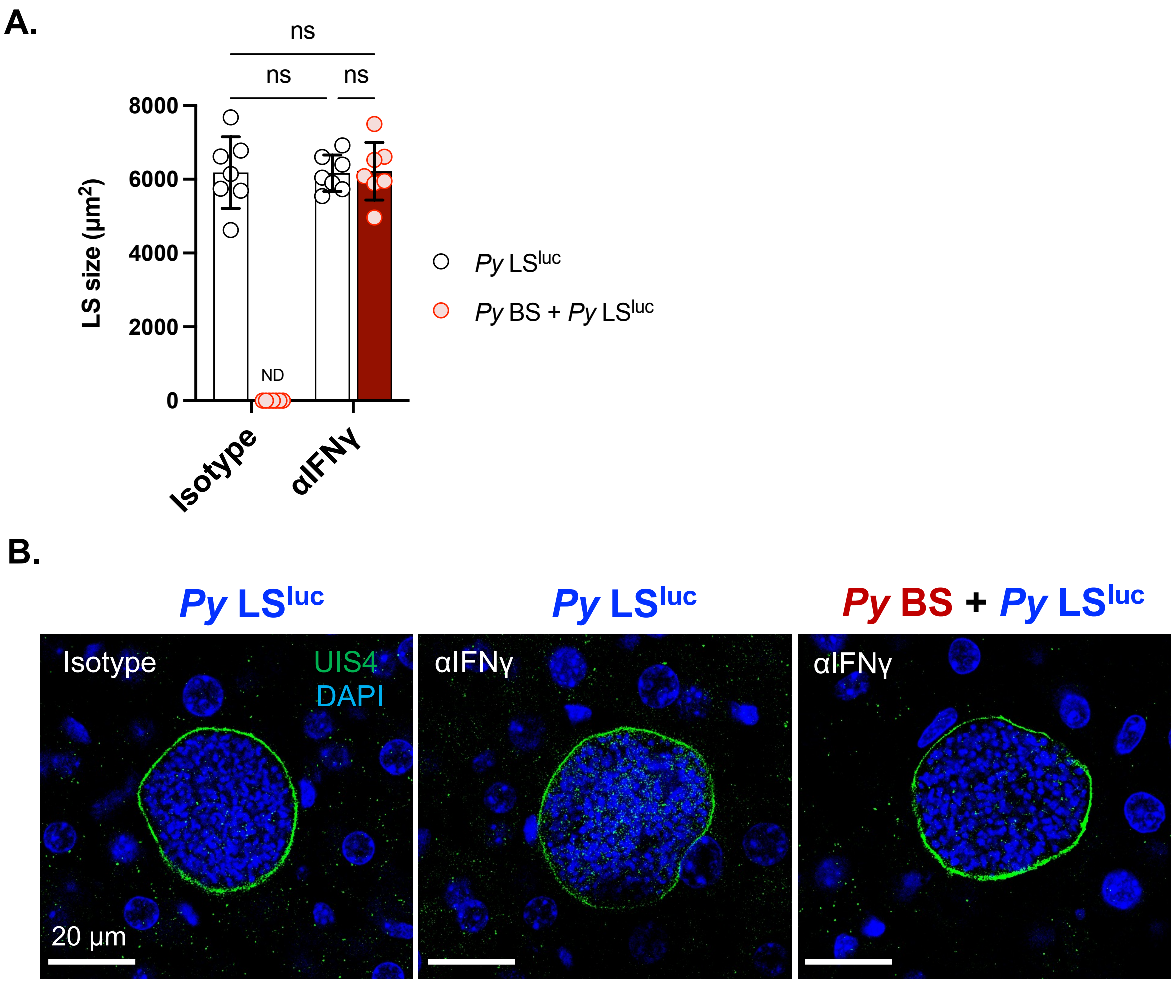

Supplementary Figure 2: ***P. yoelii BS infection suppresses LS infection via IFNγ.*** Control (*Py* LS^{luc}) and *Py* BS infected (*Py* BS + *Py* LS^{luc}) Balb/c mice were treated with Isotype or anti-IFNγ antibodies as shown in the Fig.3A experimental scheme and were infected with 50,000 wildtype *Py*$^{GFP-luc}$ sporozoites on day 4 post BS infection. Livers were collected between 43-46 hpi, tissue sections were prepared and stained with antibody against UIS4 parasite antigen and DNA staining dye, DAPI and analyzed by IFA. **(A)** Comparison of the size of late liver stage parasites (based on area at the parasite's largest circumference). At least 15 parasites were counted for each mouse from each group. ND: not detected. Two-way ANOVA with Tukey's multiple comparison test for comparing groups with two variables. Results are combined and represented as means ± SD from two independent experiments. n=7 mice per group. **(B)** The microscopic image of *Py*$^{GFP-luc}$ LS representing the size as in **(A)**. Scale bar, 20 μm. Source data are provided as a Source Data file.

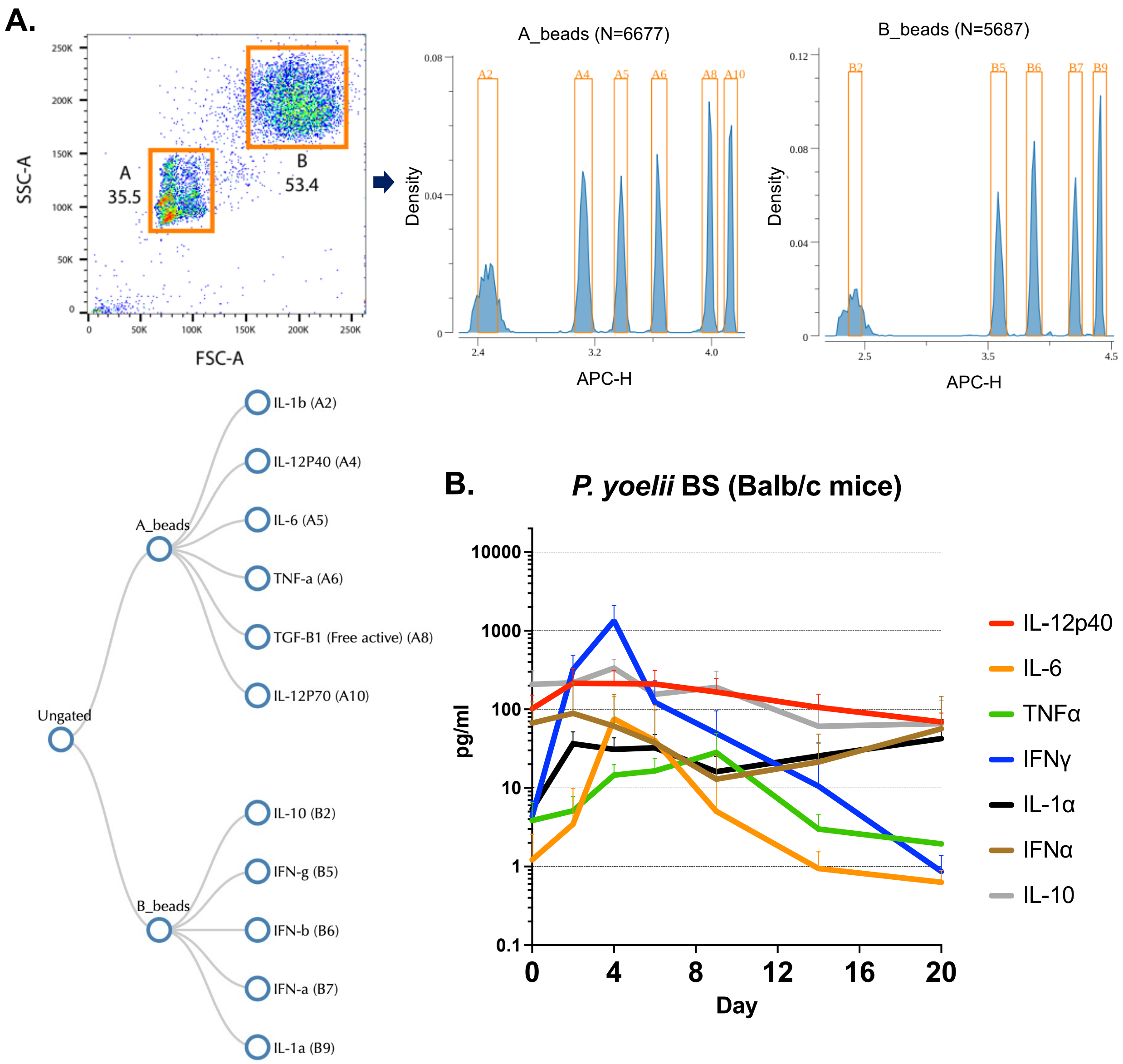

Supplementary Figure 3: ***Host-induced cytokine responses during the course of P. yoelii BS infection in Balb/c mice*** **(A)** Gating strategy. The analysis for the bead-based multiplex cytokines flow assay was done on LEGENDPlex software. The cytokines binding beads were gated based on SSC-A vs FSC-A (linear mode) to differentiate A-beads and B-beads according to their size. Each beads sets (A & B) were further gated for their internal APC fluorescence intensity to differentiate the subpopulation of beads. Then the PE fluorescence intensity was calculated for each of the subpopulation of beads which is in proportion to the amount of bound analytes. **(B)** Balb/c mice were infected with 10^6 *Py* XNL iRBCs. Blood was collected at different time points during the course of

infection and subjected to cytokines expression analysis by LEGENDplex multi-analyte flow assay. The expression of the cytokines was represented as pg/ml. Results are combined and represented as means ± SD from three independent experiments (N = 9 mice). Source data are provided as a Source Data file.

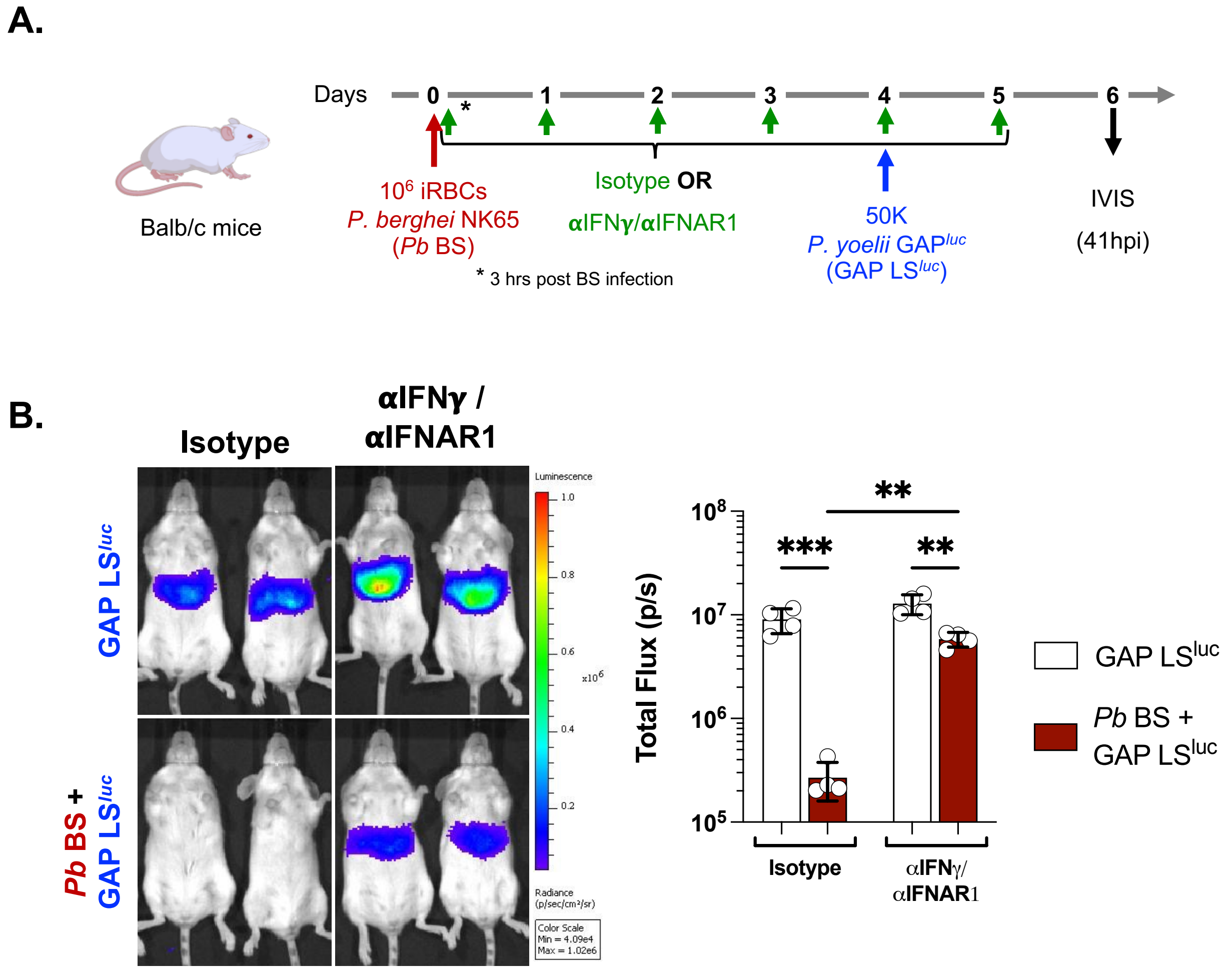

Supplementary Figure 4: ***P. berghei BS infection suppresses LS development via both type I and II interferons*** **(A)** Control (GAP LS^{luc}) and *Pb* NK65 BS infected (*Pb* BS + GAP LS^{luc}) Balb/c mice were treated with isotype or anti-IFNγ/anti-IFNAR1 monoclonal antibodies and 4 days later were infected with 50,000 *Py* GAP^{luc} sporozoites. **(B)** Parasite liver load was measured by IVIS at 43 hpi and represented as total flux (p/s). Two-way ANOVA with Tukey's multiple comparison test for comparing groups with two variables. Results are represented as means ± SD. ***P<0.01, ***P<0.001.* (N= 4 mice per group). Source data are provided as a Source Data file. The graphical illustration of the mouse in (A) was made using BioRender.com.
