# Supplementary material for: Malaria blood stage infection suppresses liver stage infection via host-induced interferons but not hepcidin
Source: Nat Commun. 2024 Mar 7;15:2104. doi: 10.1038/s41467-024-46270-3 (PMC10920859; doi:10.1038/s41467-024-46270-3)
Supplement: Supplementary file 3 — Reporting Summary [file 41467_2024_46270_MOESM3_ESM.pdf]

nature portfolio

Corresponding author(s): Stefan H.I. Kappe

Last updated by author(s): Feb 14, 2024

Reporting Summary

Nature Portfolio wishes to improve the reproducibility of the work that we publish. This form provides structure for consistency and transparency in reporting. For further information on Nature Portfolio policies, see our Editorial Policies and the Editorial Policy Checklist.

Statistics

For all statistical analyses, confirm that the following items are present in the figure legend, table legend, main text, or Methods section.

n/a	Confirmed	
☐	☒	The exact sample size (*n*) for each experimental group/condition, given as a discrete number and unit of measurement
☐	☒	A statement on whether measurements were taken from distinct samples or whether the same sample was measured repeatedly
☐	☒	The statistical test(s) used AND whether they are one- or two-sided<br>*Only common tests should be described solely by name; describe more complex techniques in the Methods section.*
☒	☐	A description of all covariates tested
☒	☐	A description of any assumptions or corrections, such as tests of normality and adjustment for multiple comparisons
☐	☒	A full description of the statistical parameters including central tendency (e.g. means) or other basic estimates (e.g. regression coefficient) AND variation (e.g. standard deviation) or associated estimates of uncertainty (e.g. confidence intervals)
☐	☒	For null hypothesis testing, the test statistic (e.g. *F*, *t*, *r*) with confidence intervals, effect sizes, degrees of freedom and *P* value noted<br>*Give P values as exact values whenever suitable.*
☒	☐	For Bayesian analysis, information on the choice of priors and Markov chain Monte Carlo settings
☒	☐	For hierarchical and complex designs, identification of the appropriate level for tests and full reporting of outcomes
☒	☐	Estimates of effect sizes (e.g. Cohen's *d*, Pearson's *r*), indicating how they were calculated

Our web collection on statistics for biologists contains articles on many of the points above.

Software and code

Policy information about availability of computer code

Data collection	IVIS data (Parasite liver stage burden) were collected on Living Image software (v4.3.1.0.15766);<br>ELISA data were collected on Gen5 software (v2.06.10);<br>qPCR data were collected on QuantStudio 5 real time PCR system;<br>Micrograph images were captured on LAS-X Life Science Microscope Software;<br>Multiplex cytokine flow data were collected on BD FACSDiva Software (v8.0.1).
Data analysis	IVIS data (Parasite liver stage burden) were analyzed on Living Image software (v4.3.1.0.15766);<br>Micrograph images were analyzed on LAS-X Life Science Microscope Software;<br>Multiplex cytokine flow data were analyzed on LEGENDplex software (v2023-02-12, 58444).<br>All the statistical data analysis and visualization was done in GraphPad Prism (v10).

For manuscripts utilizing custom algorithms or software that are central to the research but not yet described in published literature, software must be made available to editors and reviewers. We strongly encourage code deposition in a community repository (e.g. GitHub). See the Nature Portfolio guidelines for submitting code & software for further information.

Data

Policy information about availability of data

All manuscripts must include a data availability statement. This statement should provide the following information, where applicable:

- Accession codes, unique identifiers, or web links for publicly available datasets
- A description of any restrictions on data availability
- For clinical datasets or third party data, please ensure that the statement adheres to our policy

All pertinent data generated or analyzed during this study are included in this published article and its accompanying supplementary information files. The source data underlaying each figure are provided as a source data file.

Research involving human participants, their data, or biological material

Policy information about studies with human participants or human data. See also policy information about sex, gender (identity/presentation), and sexual orientation and race, ethnicity and racism.

Reporting on sex and gender	n/a
Reporting on race, ethnicity, or other socially relevant groupings	n/a
Population characteristics	n/a
Recruitment	n/a
Ethics oversight	n/a

Note that full information on the approval of the study protocol must also be provided in the manuscript.

Field-specific reporting

Please select the one below that is the best fit for your research. If you are not sure, read the appropriate sections before making your selection.

☒ Life sciences ☐ Behavioural & social sciences ☐ Ecological, evolutionary & environmental sciences

For a reference copy of the document with all sections, see nature.com/documents/nr-reporting-summary-flat.pdf

Life sciences study design

All studies must disclose on these points even when the disclosure is negative.

Sample size	No statistical methods were used to determine sample size. We had previously established in prior publications that at least 4 mice per group is required to obtain statistically robust data. As such this is the minimum number of samples per group reported in each experiment. Sample sizes are consistent with similar published studies (Minkah NK et al., Nat Commun 2019; Miller JL et al., Cell reports 2014; Sack BK et al., PloS Pathog 2015; Chora ÂF., Immunity 2023; Miller JL et al., PloS One 2013).
Data exclusions	No data points were excluded from the analysis.
Replication	All attempts of replication were successful, the observed variation is reported and contributes to the statistical calculation. Each observation was independently repeated at least once with all repeated experiments included.
Randomization	For all the experiments, the animals were randomly assigned to each group. For WT vs knockout mice comparisons, mice with similar ages and of the same sex were randomly chosen for each experiment. For experiments other than those involving mice, samples were randomly allocated. Microscopy analysis was performed in randomly acquired images.
Blinding	Investigators were not blinded to the group allocation. However, data collection was performed in an unbiased manner and the analysis were performed on quantitative endpoints that are not subject to investigator bias.

Reporting for specific materials, systems and methods

We require information from authors about some types of materials, experimental systems and methods used in many studies. Here, indicate whether each material, system or method listed is relevant to your study. If you are not sure if a list item applies to your research, read the appropriate section before selecting a response.

Materials & experimental systems

n/a	Involved in the study
☐	☒ Antibodies
☐	☒ Eukaryotic cell lines
☒	☐ Palaeontology and archaeology
☐	☒ Animals and other organisms
☒	☐ Clinical data
☒	☐ Dual use research of concern
☒	☐ Plants

Methods

n/a	Involved in the study
☒	☐ ChIP-seq
☐	☒ Flow cytometry
☒	☐ MRI-based neuroimaging

Antibodies

Antibodies used	All antibodies used in this study are detailed in the Methods sections: in "Antibodies and LDN193189", "Immunofluorescence assay (IFA) and Microscopy".<br>- anti-IL6 (clone MP5-20F3; BioXcell #BE0046),<br>- anti-IFNAR1 (clone MAR1-5A3; Leinco Technologies #I-401),<br>- anti-IFNg (clone XMG1.2; Leinco Technologies #I-1119),<br>- anti-IL12 (clone C17.8; Leinco Technologies #I-1175),<br>- anti-P. yoelii UIS4 antibody (Polyclonal; a gift from Photini Sinnis laboratory, JHU, USA, custom antibody),<br>- anti-rabbit fluorescent antibody conjugated with Alexa-fluor 594 (Polyclonal; Fisher scientific #A11012),<br>- anti-rabbit fluorescent antibody conjugated with Alexa-fluor 488 (Polyclonal; Thermofisher Scientific #A-21206)
Validation	All antibodies have been previously validated:<br>- anti-IL6 (Tsukamoto, et al., Nat Commun, 2015),<br>- anti-IFNAR1 (Sebina et al., PLoS Pathog, 2016),<br>- anti-IFNg (Sun et al. PNAS, 2012),<br>- anti-IL12 (Sun et al. PNAS, 2012),<br>- anti-P. yoelii UIS4 antibody (Schepis et al., Cell Rep, 2023),<br>- anti-rabbit fluorescent antibody conjugated with Alexa-fluor 594 (Schepis et al., Cell Rep, 2023),<br>- anti-rabbit fluorescent antibody conjugated with Alexa-fluor 488 (Schepis et al., Cell Rep, 2023)

Eukaryotic cell lines

Policy information about cell lines and Sex and Gender in Research

Cell line source(s)	HepG2-CD81 cells used in this study were originally obtained from Oliver Silvie's laboratory, Cimi, Paris, France (Silvie, O. et al., Cell Microbiol 2006)
Authentication	The cell line was not authenticated.
Mycoplasma contamination	Cells used were negative for Mycoplasma contamination.
Commonly misidentified lines (See ICLAC register)	No commonly misidentified cell lines were used in this study.

Animals and other research organisms

Policy information about studies involving animals; ARRIVE guidelines recommended for reporting animal research, and Sex and Gender in Research

Laboratory animals	For this manuscript, six to eight weeks old female Balb/c, wildtype, IFNg-/- and IFNAR1-/- C57BL/6 mice were purchased from The Jackson laboratory and eight to ten weeks old female Swiss Webster (SW) mice were purchased from Envigo laboratories. Anopheles stephensi mosquitoes were reared in the in-house insectary. Four to five days old mosquitoes were used to run the Plasmodium parasites' infection cycle.
Wild animals	No wild animals were used in this study.
Reporting on sex	Animal sex was not considered in study design.
Field-collected samples	No field-collected samples were used in this study.
Ethics oversight	All animal procedures were performed as per the regulations of the SCRI's Institutional Animal Care and Use Committee (IACUC). The animal procedures were approved by IACUC under protocols 00507 and 00666.

Note that full information on the approval of the study protocol must also be provided in the manuscript.

Flow Cytometry

Plots

Confirm that:

☒ The axis labels state the marker and fluorochrome used (e.g. CD4-FITC).

☒ The axis scales are clearly visible. Include numbers along axes only for bottom left plot of group (a 'group' is an analysis of identical markers).

☒ All plots are contour plots with outliers or pseudocolor plots.

☐ A numerical value for number of cells or percentage (with statistics) is provided.

Methodology

Sample preparation	Bead based Multiplex cytokine assay:<br>Mice were infected with Blood stage (BS) parasites by injecting 10e6 iRBCs of either Py or Pb parasite. Blood was collected in heparin tubes at different time points during the course of infection and plasma was harvested by spinning tubes at 10,000 rpm at 4oC for 10 min. The samples were immediately stored at -80oC until further use. The expression of cytokines (IFNg, IL-6, IL-12p40, IFNa, TNFa, IL-1a, IL-10, IFNb, IL-1b, IL-12p70 and TGFb) was determined in bead-based multiplex immunoassay using a customized LEGENDPlex mouse cytokine panel (Biolegend, San Diego, USA). The assay was performed as per manufacturer’s instructions and data was acquired on BD LSR II flow cytometer. The standard curve was created for each analyte (R-square >0.99) by performing 1:4 dilution (8-points) of mouse custom standard panel (provided in the kit) with starting top concentration 10,000 pg/ml or 50,000 pg/ml (IFNb).<br><br>No flow cytometry analysis was performed on any cell population in this manuscript, except for the bead based cytokines detection in the mouse blood using customized LEGENDPlex kit from Biolegend. A numerical value for number of cells do not apply for fluorescence bead based analysis.
Instrument	BD FACS LSR II analyzer
Software	Multiplex cytokine flow data were collected on BD FACSDiva Software (v8.0.1) and analyzed on LEGENDplex software (v2023-02-12, 58444).
Cell population abundance	Not applicable. No cell analysis or cell sorting was performed.
Gating strategy	The analysis was done on LEGENDPlex software according to manufacturer's manual. The cytokines binding beads were gated based on SSC-A vs FSC-A (linear mode) to differentiate A-beads and B-beads according to their size. Each beads sets (A & B) were further gated for their internal APC fluorescence intensity to differentiate the subpopulation of beads. Then the PE fluorescence intensity was calculated for each of the subpopulation of beads which is in proportion to the amount of bound analytes.

☒ Tick this box to confirm that a figure exemplifying the gating strategy is provided in the Supplementary Information.
